# Supplementary material for: Sorting at embryonic boundaries requires high heterotypic interfacial tension
Source: Nat Commun. 2017 Jul 31;8:157. doi: 10.1038/s41467-017-00146-x (PMC5537356; doi:10.1038/s41467-017-00146-x)
Supplement: Supplementary file 2 — Supplementary Software 1 [file 41467_2017_146_MOESM2_ESM.zip › PottsModel/SrcPottsModel/doc/index-files/index-15.html]

O-Index


JavaScript is disabled on your browser.


Skip navigation links


- Overview
- Package
- Class
- Use
- Tree
- Deprecated
- Index
- Help

- Prev Letter
- Next Letter

- Frames
- No Frames

- All Classes

A B C D E F G H I J K L M N O P R S T U V W 


## O

observe(PottsEngine.State) - Method in class engine.AreaEnergyStatistic


observe(PottsEngine.State) - Method in class engine.AreaStatistic


observe(PottsEngine.State) - Method in class engine.CellEnergyStatistics


observe(PottsEngine.State) - Method in class engine.CSVStatistic


observe(PottsEngine.State) - Method in class engine.DispersionIndex


observe(PottsEngine.State) - Method in class engine.EnergyStatistic


observe(PottsEngine.State) - Method in class engine.HBLStatistic


observe(PottsEngine.State) - Method in class engine.HMDStatistic


observe(PottsEngine.State) - Method in class engine.InteractionEnergyStatistic


observe(PottsEngine.State) - Method in class engine.IsoperimetricQuotientStatistic


observe(PottsEngine.State) - Method in class engine.PerimeterStatistic


observe(PottsEngine.State) - Method in class engine.Statistic
:   Observe values during MCS.

observe(PottsEngine.State) - Method in class engine.TypeSpecificAreaStatistic


observe(PottsEngine.State) - Method in class engine.TypeSpecificNearestNeighborStatistic


observe(PottsEngine.State) - Method in class engine.TypeSpecificNumNeighborsStatistic


observe(PottsEngine.State) - Method in class engine.TypeSpecificPerimeterStatistic


observe(PottsEngine.State) - Method in class engine.TypeSpecificStatistic


observe(Statistic, double, double, String) - Method in class gui.PlotPanel


Observer - Interface in mvc
:   Interface that can be implemented to provide observer support.

OffsetCoordinates(double, double) - Constructor for class gui.Hexagon.OffsetCoordinates

A B C D E F G H I J K L M N O P R S T U V W

Skip navigation links


- Overview
- Package
- Class
- Use
- Tree
- Deprecated
- Index
- Help

- Prev Letter
- Next Letter

- Frames
- No Frames

- All Classes
